# Associations Between Maternal Helminth and Malaria Infections in Pregnancy and Clinical Malaria in the Offspring: A Birth Cohort in Entebbe, Uganda

**DOI:** 10.1093/infdis/jit397

**Published:** 2013-07-31

**Authors:** Juliet Ndibazza, Emily L. Webb, Swaib Lule, Harriet Mpairwe, Miriam Akello, Gloria Oduru, Moses Kizza, Helen Akurut, Lawrence Muhangi, Pascal Magnussen, Birgitte Vennervald, Alison Elliott

**Affiliations:** 1 MRC/UVRI Uganda Research Unit on AIDS; 2 Entebbe Hospital, Entebbe, Uganda; 3 London School of Hygiene and Tropical Medicine, United Kingdom; 4 Institute for International Health, Immunology and Microbiology, Faculty of Health and Medical Sciences; 5 Section for Parasitology and Aquatic Diseases, Faculty of Health and Medical Sciences, Copenhagen University, Denmark

**Keywords:** malaria, helminths, coinfections, pregnancy, childhood

## Abstract

***Background.*** Helminth and malaria coinfections are common in the tropics. We investigated the hypothesis that prenatal exposure to these parasites might influence susceptibility to malaria in childhood.

***Methods.*** In a birth cohort of 2345 mother–child pairs in Uganda, maternal helminth and malaria infection status was determined during pregnancy, and childhood malaria episodes were recorded from birth to age 5 years. We examined associations between maternal infections and malaria in the offspring.

***Results.*** Common maternal infections were hookworm (45%), *Mansonella perstans* (21%), *Schistosoma mansoni* (18%), and *Plasmodium falciparum* (11%). At age 5 years, 69% of the children were still under follow-up. The incidence of malaria was 34 episodes per 100 child-years, and the mean prevalence of asymptomatic malaria at annual visits was 5.4%. Maternal hookworm and *M. perstans* infections were associated with an increased rate of childhood clinical malaria (adjusted hazard ratio [aHR], 1.24, 95% confidence interval [CI], 1.10–1.41; aHR, 1.20, 95% CI, 1.05–1.38, respectively). *S. mansoni* infection had no consistent association with childhood malaria.

***Conclusions.*** This is the first report of an association between helminth infections in pregnancy and malaria in the offspring and indicates that helminth infections in pregnancy may increase the burden of childhood malaria morbidity.

Chronic infection with *Plasmodium* and helminths causes an enormous public health burden in the tropics [1–5]. Malaria in pregnancy has been associated with increased risk of maternal anemia, low birth weight, stillbirth, and maternal death [6, 7]. Recent evidence suggests that prenatal exposure to *Plasmodium falciparum* may increase malaria risk in early childhood [8, 9]; however, little is known about the long-term consequences for young children.

In the 1990s, estimates suggested that 44 million of the world's pregnant women harbored hookworm [10], and it was suggested that helminth infections might be particularly detrimental to the mother during pregnancy [11, 12]. However, in the Entebbe Mother and Baby Study (EMaBS) we found unexpectedly little association between maternal helminths and maternal anemia and none of the expected benefits of anthelminthic treatment in pregnancy for birth outcomes [13, 14]. Still, there is a dearth of literature on the consequences of helminth infections in pregnancy for the child, and effects of prenatal exposure to malaria*–*helminth coinfections on childhood malaria have not been addressed.

Studies including the EMaBS have reported associations between malaria and helminth infections in pregnant women [15, 16], and others have reported associations in children [17, 18]. Hookworm infection has been related to increased susceptibility to malaria infection in pregnancy [2, 16, 19] and in children [2, 20]. However, results have been inconsistent [15, 21]. Immunoregulatory mechanisms have been proposed. Helminth infections induce T-cell hyporesponsiveness, downmodulating immunity to their own as well as other antigens [22–25], and *Plasmodia* may possibly modulate responses to helminth coinfections [26]. Prenatal exposure to pathogen antigens might enhance fetal tolerance or sensitization to the antigens, leading to a failure to mount a response to the infection [9] or the development of infection resistance [27], respectively. Whether such immunological effects have a measurable impact on the incidence of malaria is uncertain [28, 29].

In the EMaBS, treatment with anthelminthics in pregnancy had no effect on childhood malaria incidence. However, we speculated that the single-dose treatment might be insufficient to change an effect of helminths established earlier during pregnancy. This study used EMaBS data (ISRCTN32849447) [30] to examine associations between helminth and malaria infections in pregnancy and malaria in the offspring.

## METHODS

The study was approved by the Science and Ethics Committee of the Uganda Virus Research Institute, the Uganda National Council for Science and Technology and the ethics committee of the London School of Hygiene and Tropical Medicine. Project staff explained the study to the participants in the local language and provided participants with a study information letter to take home. Written informed consent was obtained from the mother during pregnancy and from the mother or caregiver when the child was aged 1 year.

### Study Population

The study area is on the northern shores of Lake Victoria in Uganda, a high malaria transmission area. *P. falciparum* is the dominant *Plasmodium* species; *Anopheles gambiae* and *Anopheles fenestus* are the dominant vectors [31]. The area consists of urban, rural, and fishing communities.

### Study Design

The EMaBS enrolled 2507 pregnant women in their second or third trimester between April 2003 and November 2005; 2345 live births were recorded. Inclusion and exclusion criteria are described elsewhere [30]. We present an observational analysis of the trial cohort.

At enrolment, the median gestational age was 27 weeks (interquartile range, 22–31). Before receiving the trial intervention, women gave a blood and stool sample for assessment of parasite infections. Women were then randomized to single-dose albendazole (400 mg) or placebo, and praziquantel (40 mg/kg) or placebo; their offspring were randomized to receive quarterly single-dose albendazole (200 mg from age 15 to 21 months and 400 mg from age 24 to 60 months) or placebo. Ferrous sulphate was provided monthly, and intermittent presumptive treatment for malaria (sulphadoxine-pyrimethamine) twice during the pregnancy. All women received anthelminthic treatment 6 weeks after delivery. After delivery, mothers were invited to bring the children to the research clinic for routine immunizations and any illness and for quarterly study visits to age 5 years. Community workers visited the children fortnightly and referred sick children to the clinic. Clinical malaria episodes were recorded prospectively. At annual scheduled visits, the children were examined for *P. falciparum* and helminth infections and were treated according to clinical guidelines if infections were found.

Participants' addresses at enrollment were geo-referenced using handheld GPS receivers, and geographical zones were established based on features such as coastline, forest, location of settlements, and altitude [16].

### Diagnosis of Infections

Women provided blood and stool samples at enrollment and after delivery; children provided samples at illness and routine annual visits. Blood samples were examined for *Mansonella perstans* using the modified Knott's method [32]. Thick blood films were stained with Leishman's stain, malaria parasites were counted against 200 leucocytes, and at least 100 high-power fields were examined before a film was declared negative. Duplicate Kato–Katz slides were prepared and examined within 30 minutes for hookworm and the following day for other helminth eggs [33, 34]. Human immunodeficiency virus (HIV) serology was performed for mothers and children aged ≥18 months using a rapid test algorithm [13]; for infants, RNA and DNA polymerase chain reaction methods were used. Vector Control Division, Ministry of Health, Uganda, provided quality control for Kato–Katz slides, and the Medical Research Council Laboratories at Uganda Virus Research Institute provide quality control for malaria films.

### Statistical Methods

The aim was to examine the association between maternal helminth and malaria infections in pregnancy and malaria in the offspring. The sample size for the study was determined for the original trial objectives. To test the hypothesis that maternal albendazole or praziquantel in pregnancy would influence the incidence of malaria in infancy (assumed to be 0.5 per person-year in the maternal placebo group), a study with 2500 participants would have 80% power to show an 18% reduction or a 19% increase in the incidence of malaria in infancy, with *P* < .05, assuming that each intervention had an independent effect. Either direction of effect could happen, depending on whether helminth coinfection increases susceptibility to malaria infection and disease or decreases inflammation and hence reduces malaria-induced morbidity.

Of 2345 live births, 53 twins and 3 triplets were excluded, leaving 2289 children for inclusion in the analysis. Primary outcome was incidence of childhood clinical malaria from birth to age 5 years, and secondary outcome was prevalence of asymptomatic *P. falciparum* parasitemia as determined at annual visits to age 5 years. Childhood clinical malaria was defined as a history of recent fever or axillary temperature of ≥37.5°C and any parasitemia. Asymptomatic *P. falciparum* parasitemia was defined as a positive malaria slide in the absence of fever on the sampling day. Key predictor variables were maternal *P. falciparum*, hookworm, *M. perstans,* and *S. mansoni* infections at enrollment*.* Malaria was defined as peripheral parasitemia.

For the primary outcome, time at risk began at birth and was censored at loss to follow-up, death, or age 5 years. Clinical malaria episodes within 14 days of an initial presentation were regarded as a recrudescence and excluded from the analysis; time at risk was adjusted accordingly, excluding these 14-day periods from the total person-time denominator. Crude hazard ratios (HRs) for the effect of malaria and helminth infections in pregnancy on the incidence of childhood malaria were calculated using Cox regression with robust standard errors to allow for within-child clustering of malaria episodes. Independent risk factors for maternal infections and childhood malaria that were significant (*P* ≤ .10) at the univariable analyses were entered into multivariable models. Variables included in the models were maternal age, education, parity, HIV status, mosquito net ownership, socioeconomic status, and geographical residential zone. Maternal *P. falciparum* and HIV infections and child albendazole were assessed as potential effect modifiers of the association between each maternal infection and childhood malaria. The secondary outcome was analyzed by combining data from all annual visits and comparing repeated prevalence of parasitemia between maternal malaria and helminth infection groups using random effects logistic regression, adjusting for the same confounders. Adjusted *P* values were calculated using likelihood ratio tests. Albendazole and praziquantel treatment in pregnancy had no effect on the incidence of clinical malaria [35]; hence there was no need to allow for them in the analysis. Statistical analysis was performed using Stata version 11. Rather than formally adjusting for multiple testing, we interpreted consistent results for related outcomes as providing evidence of a true association. Significance was defined as *P* values ≤ .05.

## RESULTS

Of 2345 live births, 1622 children (69%) were still under follow-up at 5 years, and a total of 33 178 clinic visits for illnesses were recorded [35]. A trial flow chart has been reported previously [35] and is available as Supplementary Figure 1. The total number of clinic visits was similar across maternal helminth groups (data not shown). Table 1 shows characteristics of the participating women and children. A complete description of maternal infections in pregnancy, previously reported [36], is provided in Supplementary Appendix 1. The overall mean *P. falciparum* parasite count in pregnancy was 163 (standard deviation [SD], 357), and only 51 (22%) of 236 mothers with parasitemia had >1000 parasite/µL blood. Two hundred two (8%) women were infected with *P. falciparum* and at least one of hookworm or *S. mansoni* or *M. perstans*. At delivery, only 69 of 2133 (3%) of the women tested had malaria parasitemia. Maternal HIV prevalence was 11%.

**Table 1.** Characteristics of Mothers and Children Who Participated in the Study

Group	Characteristic	Summary
Mothers (n = 2289)	Mean age (±SD) at enrollment Gravidity	24 (5.4)
	1	611 (27%)
	2–4	1307 (57%)
	≥5	371 (16%)
	Trimester (4 mv)	
	2	1051 (46%)
	3	1234 (54%)
	Highest educational level attained (4mv)	
	None	81 (4%)
	Primary	1152 (50%)
	Secondary	860 (38%)
	Tertiary	192 (8%)
	Socioeconomic status (44 mv)	
	Lower	1028 (45%)
	Higher	1217 (53%)
	Infections	
	Any helminth (29 mv)	1545 (68%)
	Hookworm (9 mv)	1004 (45%)
	*Schistosoma mansoni* (9 mv)	415 (18%)
	*Mansonella perstans* (8 mv)	492 (21%)
	*Plasmodium falciparum* (43 mv)	236 (10%)
	HIV	261 (11%)
	Received IPTp	2211 (97%)
	Owns mosquito net	1131 (49%)
	Primary source of water (5 mv)	
	Open source	1910 (83%)
	Piped source	374 (16%)
	Primary source of fuel (6 mv)	
	Firewood	408 (18%)
	Charcoal	1626 (71%)
	Paraffin	49 (2%)
	Gas/electricity	200 (9%)
Children (n = 2289)	Male	1167 (51%)
	Mean birthweight (±SD)	3.19 (±0.49)

Abbreviations: HIV, human immunodeficiency virus; IPTp, intermittent presumptive treatment for malaria; mv, missing values; SD, standard deviation.

The prevalence of helminth infections was low among the children: 2.9% (95% confidence interval [CI], 2.0–3.8), 5.2% (95% CI, 4.0–6.3), 7.7% (95% CI, 6.3–9.1), 9.0% (95% CI, 7.4–10.5), and 9.5% (95% CI, 7.9–11.1) at annual visits 1, 2, 3, 4, and 5 years, respectively. Of 2289 children, 1161 (51%) had at least 1 malaria episode; 459 (20%) had 1 episode, and 702 (31%) had ≥2 episodes (18 children had >10 episodes). The overall malaria incidence rate was 34 per 100 child-years, higher in the first 2 years (41 and 53 per 100 child-years, respectively), than in years 3, 4, and 5 (31, 20, and 20 per 100 child-years, respectively). The annual prevalence of asymptomatic parasitemia among the children was 5.9% (95% CI, 4.8–7.2), 7.1% (95% CI, 5.9–8.5), 4.7% 95% CI, (3.7–6.0), 4.3% (95% CI, 3.3–5.6) and 4.8% (95% CI, 3.8–6.1) at 1, 2, 3, 4, and 5 years, respectively. The overall mean parasite density was 8292 parasites/µL (SD, 18 576).

### Variables Associated With Helminth and Malaria Infections in Pregnancy

Older age, higher education, higher socioeconomic status, and mosquito net ownership were associated with a lower prevalence of maternal helminth and malaria infections [37]. HIV infection was negatively associated with hookworm, whereas HIV, hookworm, and *M. perstans* infections were associated with increased odds of maternal malaria. The risk of maternal helminth and malaria infection in pregnancy varied significantly by geographical zone [16].

### Variables Associated With Childhood Malaria

Table 2 shows that children of younger, poorer mothers, who had a low education level and did not own a mosquito net had a higher risk of childhood clinical malaria. The risk also varied significantly by both parity and geographical location of residence.

**Table 2.** Variables Associated With Clinical Malaria Episodes in Childhood (aged 0–5 years)

Maternal Risk Factor	Incidence Rate per 100 Child-Years (95% CI)	Crude HR (95% CI)	Adjusted HR^a^ (95% CI)	*P* Value
Age (years)				
<25	34.70 (33.21–36.26)	1	1	
≥25	32.57 (30.79–34.45)	0.94 (.83–1.08)	0.84 (.71–.99)	.04
Parity				
Primipara	31.78 (29.60–34.11)	1	1	
Multipara (2–4)	32.83 (31.35–34.39)	1.04 (.90–1.20)	1.13 (.97–1.30)	
Grand multipara ( ≥5)	40.55 (37.53–43.80)	1.29 (1.06–1.57)	1.40 (1.10–1.78)	.03
HIV				
Negative	34.38 (33.15–35.64)	1	1	
Positive	29.21 (25.96–32.86)	0.85 (.68–1.06)	0.82 (.67–1.01)	.14
Socioeconomic				
Lower status	37.57 (35.76–39.48)	1	1	
Higher status	30.30 (28.82–31.85)	0.81 (.71–.92)	0.85 (.75–.96)	.01
Education				
None/Primary	39.01 (37.32–40.78)	1	1	
Postprimary	28.12 (26.60–29.73)	0.73 (.64–.82)	0.83 (.73–.94)	.003
Owns bed net				
Yes	27.00 (25.55–28.53)	1	1	.01
No	40.67 (38.90–42.52)	1.50 (1.32–1.71)	1.17 (1.04–1.33)	
Water source				
Piped	29.93 (28.75–31.17)	1	1	
Open	53.10 (49.63–56.82)	1.78 (1.54–2.06)	1.39 (1.19–1.61)	<.001
Fuel source				
Indoor (electricity/gas)	14.30 (11.92–17.16)	1	1	
Outdoor (paraffin/charcoal/wood)	35.79 (34.55–37.08)	2.53 (2.01–3.18)	1.83 (1.44–2.32)	<.001
Geographical zone				
1	15.19 (13.04–17.69)	1	1	
2	19.32 (16.87–22.13)	1.26 (.93–1.71)	1.57 (1.15–2.13)	<.001
3	46.98 (44.00–50.18)	3.07 (2.39–3.95)	3.28 (2.54–4.25)	
4	16.49 (13.98–19.45)	1.08 (.79–1.48)	1.28 (.93–1.76)	
5	28.47 (25.78–31.43)	1.85 (1.42–2.40)	2.14 (1.63–2.80)	
6	38.72 (27.86–30.78)	2.52 (1.91–3.33)	2.60 (1.96–3.44)	
7	38.71 (33.91–44.19)	2.54 (1.83–3.52)	2.63 (1.90–3.65)	
8	32.95 (29.02–37.42)	2.17 (1.60–2.95)	2.21 (1.63–3.01)	
9	64.60 (58.58–71.24)	4.23 (3.22–5.57)	3.96 (3.00–5.22)	
10	30.38 (23.15–39.87)	2.00 (1.15–3.47)	1.99 (1.14–3.44)	
11	67.79 (54.52–84.28)	4.42 (2.72–7.18)	3.67 (2.21–6.10)	
12	57.00 (38.21–85.05)	3.63 (1.23–10.73)	1.04 (.28–3.82)	
13	77.28 (63.46–94.11)	5.12 (3.64–7.20)	3.57 (2.46–5.17)	

Abbreviations: CI, confidence interval; HIV, human immunodeficiency virus; HR, hazard ratio.

^a^ Variables adjusted for each other.

### Association Between Malaria in Pregnancy and Childhood Malaria

After adjusting for maternal age, parity, education, mosquito net ownership, household socioeconomic status, maternal HIV status, and location of residence, maternal malaria was associated with a significantly higher incidence of childhood malaria (adjusted hazard ratio [aHR], 1.23; 95% CI, 1.01–1.51]; *P* = .04). However, the positive association observed between maternal malaria and childhood asymptomatic parasitemia did not reach statistical significance (adjusted odds ratio [aOR], 1.27; 95% CI, .83–1.97; *P* = .28). Infection intensity did not affect the association. The association was not modified by maternal HIV (*P*_interaction_ = .20) or child albendazole (*P* = .60).

### Association Between Maternal Helminth Infections in Pregnancy and Childhood Malaria

Children of mothers with hookworm or *M. perstans* in pregnancy had significantly higher rates of clinical malaria and increased odds of asymptomatic parasitemia compared with children of uninfected mothers (Tables 3 and 4). Simultaneously adjusting for each helminth did not change the association between maternal hookworm and childhood clinical malaria (aHR, 1.18; 95% CI, 1.04–1.34; *P* = .01) or childhood asymptomatic parasitemia (aOR, 1.43; 95% CI, 1.08–1.90; *P* = .01) but weakened the association between maternal *M. perstans* and childhood clinical malaria (aHR, 1.14; 95% CI, 1.00–1.30; *P* = .06) or childhood asymptomatic parasitemia (aOR, 1.26; 95% CI, .92–1.74; *P* = .15). Overall, there was no association between maternal *S. mansoni* infection and childhood clinical malaria or asymptomatic parasitaemia (Tables 3 and 4). Maternal HIV did not modify the associations between childhood malaria and maternal hookworm (*P* = .10), *M. perstans* (*P* = .80), or *S. mansoni* (*P* = .80). Similarly, child albendazole did not modify the associations between childhood malaria and maternal hookworm (*P* = .30), *M. perstans* (*P* = .10), or *S. mansoni* (*P* = .70).

**Table 3.** Association Between Maternal Helminth Infections and Clinical Malaria Episodes in Childhood (aged 0–5 years)

Maternal Infection Status	Incidence Rate per 100 Person-Years (95% CI)	Cox HR (95% CI)	Adjusted HR^a^ (95% CI)	*P* Value	Adjusted HR^b^ (95% CI)	*P* Value
No hookworm	29.28 (27.86–30.78)	1	1		1	
Hookworm	39.93 (38.04–41.91)	1.36 (1.20–1.54)	1.26 (1.10–1.43)	<.001	1.23 (1.09–1.40)	.001
No *Mansonella perstans*	31.83 (30.58–33.15)	1	1		1	
*M. perstans*	40.75 (38.06–43.64)	1.30 (1.12–1.51)	1.24 (1.07–1.42)	.003	1.19 (1.03–1.37)	.02
No *Schistosoma mansoni*	33.07 (31.81–34.38)	1	1		1	
*S. mansoni*	37.74 (34.96–40.74)	1.14 (.97–1.35)	1.07 (.91–1.26)	.43	1.06 (.90–1.25)	.48

Abbreviations: CI, confidence interval; HR, hazard ratio.

^a^ Adjusted for maternal age, education, parity, net ownership, socioeconomic status, and maternal malaria and human immunodeficiency virus infections.

^b^ In addition adjusted for geographical zone.

**Table 4.** Association Between Maternal Helminth Infections in Pregnancy and the Prevalence of Childhood Asymptomatic Parasitemia (aged 0–5 years)

Maternal Infection Status	No. of Children Ever Parasitemic at Any Time Point (%)	Crude OR (95% CI)	Adjusted OR^a^ (95% CI)	*P* Value	Adjusted OR^b^ (95% CI)	*P* Value
No hookworm	160 (4.2)	1	1		1	
Hookworm	179 (6.6)	1.76 (1.31–2.38)	1.63 (1.22–2.17)	.001	1.57 (1.18–2.08)	.002
No *Mansonella perstans*	282 (4.9)	1	1		1	
*M. perstans*	113 (7.4)	1.71 (1.24–2.35)	1.49 (1.08–2.06)	.02	1.36 (.99–1.88)	.06
No *Schistosoma mansoni*	322 (5.4)		1		1	
*S. mansoni*	76 (5.7)	1.07 (.75–1.53)	1.00 (.69–1.44)	1.00	0.99 (.69–1.41)	.96

Abbreviations: CI, confidence interval; OR, odds ratio.

^a^ Adjusted for maternal age, education, parity, net ownership, socioeconomic status, and maternal malaria and human immunodeficiency virus infection.

^b^ In addition adjusted for geographical zone.

### Association Between Malaria–Helminth Coinfections in Pregnancy and Childhood Malaria

Associations between childhood malaria and maternal hookworm or *M. perstans* did not differ when stratified by maternal malaria status. An association between maternal *S. mansoni* and childhood parasitemia was observed only in the presence of maternal malaria (Tables 5 and 6).

**Table 5.** Association Between Maternal Helminth Infections in Pregnancy and Childhood Clinical Malaria (aged 0–5 years), Stratified by Maternal Malaria

Maternal Infection Status	Incidence rate per 100 pyrs (95% CI)	Cox HR (95% CI)	Adjusted HR^a^ (95% CI)	*P* Value	Adjusted HR^b^ (95% CI)	*P* Value
No malaria						
No hookworm	28.81 (27.33,30.38)	1	1		1	
Hookworm	37.93 (35.95,40.01)	1.31 (1.15–1.50)	1.20 (1.05–1.38)	.01	1.17 (1.03–1.34)	.02
Had malaria						
No hookworm	35.09 (29.96,41.09)	1	1		1	
Hookworm	52.46 (46.48,59.21)	1.48 (1.03–2.14)	1.63 (1.15–2.32)	.01	1.60 (1.13–2.26)	.01
*P*_interaction_ = .23^c^						
No malaria						
No *Mansonella perstans*	30.79 (29.49,32.15)	1	1		1	
*M. perstans*	40.05 (37.13,43.21)	1.30 (1.12–1.51)	1.26 (1.08–1.46)	.003	1.21 (1.03–1.40)	.02
Had malaria						
No *M. perstans*	44.33 (39.35,49.93)	1	1		1	
*M. perstans*	44.34 (37.68,52.18)	0.99 (.70–1.40)	1.25 (.87–1.79)	.23	1.22 (.85–1.76)	.28
*P*_interaction_ = .32^c^						
No malaria						
No *Schistosoma mansoni*	31.85 (30.53,33.22)	1	1		1	
*S. mansoni*	36.41 (33.52,39,54)	1.14 (.96–1.36)	1.10 (.92–1.31)	.29	1.09 (.92–1.29)	.33
Had malaria						
No *S. mansoni*	43.91 (39.49,48.83)	1	1		1	
*S. mansoni*	46.36 (36.97,58.14)	1.06 (.61–1.86)	0.86 (.53–1.41)	.56	0.88 (.53–1.44)	.60
*P*_interaction_ = .50^c^						

Abbreviations: CI, confidence interval; HR, hazard ratio.

^a^ Adjusted for maternal age, education, parity, net ownership, socioeconomic status, and maternal human immunodeficiency virus infection.

^b^ In addition adjusted for geographical zone.

^c^ Interaction test corresponding to the model adjusted for geographical zone.

**Table 6.** Association Between Maternal Helminth Infections in Pregnancy and the Prevalence of Childhood Asymptomatic Parasitemia (aged 0–5 years), Stratified by Maternal Malaria

Maternal Infection Status	No. of Children Ever Parasitemic at Any Time Point (%)	Crude OR (95% CI)	Adjusted OR^b^ (95% CI)	*P* Value	Adjusted OR^c^ (95% CI)	*P* Value
No malaria						
No hookworm	160 (4.2)	1	1		1	
Hookworm	179 (6.6)	1.76 (1.31–2.38)	1.48 (1.09–2.00)	.01	1.41 (1.05–1.90)	.02
Had malaria						
No hookworm	15 (4.5)	1	1		1	
Hookworm	38 (10.4)	2.70 (1.14–6.44)	3.38 (1.33–8.63)	.01	3.29 (1.30–8.34)	.01
*P*_interaction_ = .11^a^						
No malaria						
No *Mansonella perstans*	249 (4.7)	1	1		1	
*M. perstans*	90 (7.0)	1.63 (1.15–2.31)	1.44 (1.02–2.04)	.04	1.31 (.93–1.84)	.12
Had malaria						
No *M. perstans*	31 (6.7)	1	1		1	
*M. perstans*	22 (9.3)	1.62 (.67–3.90)	2.17 (.80–5.88)	.13	2.09 (.78–5.65)	.15
*P*_interaction_ = .77^a^						
No malaria						
No *Schistosoma mansoni*	279 (5.3)	1	1		1	
*S. mansoni*	60 (5.0)	0.95 (.64–1.40)	0.85 (.57–1.26)	.42	0.84 (.57–1.23)	.36
Had malaria						
No *S. mansoni*	37 (6.3)	1	1		1	
*S. mansoni*	16 (14.2)	2.98 (1.08–8.24)	3.12 (1.05–9.27)	.04	3.15 (1.06–9.39)	.04
*P*_interaction_ = .02^a^						

Abbreviations: CI, confidence interval; OR, odds ratio.

^a^ Interaction test corresponding to the model adjusted for geographical zone.

^b^ Adjusted for maternal age, education, parity, net ownership, socioeconomic status, and maternal human immunodeficiency virus infection.

^c^ In addition adjusted for geographical zone.

## DISCUSSION

To our knowledge, this is the first report of a birth cohort showing an association between helminth infections in pregnancy and childhood malaria. Earlier studies exploring the influence of helminth infections on the course of malaria and the effect of malaria–helminth coinfections [18, 38–41] used different study designs and showed both beneficial and detrimental association [36].

Our main finding was higher malaria morbidity (both in terms of clinical episodes and of asymptomatic parasitemia) among children of mothers with hookworm and *M. perstans* infections in pregnancy compared with children of uninfected mothers.

Additionally we observed an increased rate of childhood clinical malaria in children of mothers with malaria compared with children of mothers without malaria. Whereas other studies have described associations between placental malaria infection and childhood malaria [8, 9, 42, 43], we report associations with maternal peripheral parasitemia.

At enrolment, pregnant women with hookworm and *M. perstans* infections were at increased risk of peripheral malaria parasitemia [16], suggesting that the association we observed between the maternal helminth infections and childhood malaria might be explained by the association with malaria in pregnancy. However, in the analyses stratified by maternal malaria status, maternal hookworm infection was associated with an increased rate of childhood clinical malaria and an increased prevalence of childhood parasitemia, irrespective of whether the mother had malaria or not. Also, the association between maternal hookworm and childhood malaria remained consistent after simultaneously adjusting for each helminth, whereas the adjustment weakened the association between maternal *M. perstans* and childhood malaria. This suggests that the association between maternal hookworm and childhood malaria may be independent of the association with maternal malaria and the other helminth infections.

A possible explanation for these findings is that mothers with helminths and malaria in pregnancy came from a high malaria transmission environment and that the child's increased malaria risk was partly due to this. The risk of maternal malaria varied significantly by geographical zone of residence [16], suggesting that children of women living in high malaria transmission areas would be more exposed than children of mothers living in areas with lower transmission. Nevertheless, the association between maternal malaria, hookworm, and *M. perstans* and childhood malaria persisted after adjusting for location of residence. However, we were unable to accurately assess the contribution of malaria transmission in the observed associations because we did not measure malaria exposure at the household or individual level. In the multivariable analyses, we adjusted for geographical location of residence assuming homogenous malaria transmission zones within an area of 4 km in diameter, which may not be sensitive to within-area transmission variations, and therefore we cannot exclude a role for malaria transmission in the observed associations.

Previous studies have reported greater malaria morbidity associated with *S. mansoni* coinfection [44]. In our study, maternal schistosome infections were mostly light to moderate, and only 2% (37 of 2237) of the mothers had malaria–*S. mansoni* coinfection, but we observed that children of *S. mansoni–*infected mothers were at higher risk of malaria parasitemia only if the mother also had malaria. This result should be interpreted with caution because it is not consistent with the association between clinical malaria and maternal *S. mansoni,* and we cannot rule out that this finding was due to chance alone. However, the result suggests the hypothesis that exposure to both malaria and helminths is required to alter the way the fetus's initial response to malaria is primed. In fact this may also be the case for the malaria–hookworm interaction. Although the associations between maternal hookworm and childhood malaria were statistically similar between children of mothers with and without malaria, the point estimates were lower in the strata of mothers without malaria. There may have been some misclassification of maternal malaria and, if exposure of the fetus to both malaria and hookworm is required for the observed effects, the observed trend in hazard ratios could have occurred if about 20% of the “no malaria” mothers actually had malaria at some time during the pregnancy. This would explain the absence of interaction between maternal malaria and hookworm or *M. perstans*.

A possible immunological explanation for the observed results is that fetal exposure to maternal helminth and malaria infections may induce T-cell hyporesponsiveness, downmodulating immunity to helminths [24] and malaria antigens and modifying fetal acquisition of immunity to malaria [9, 21, 45]. Tolerance in offspring exposed to parasite antigens in utero [46], resulting in increased susceptibility to infection, has been reported. Alternatively, coexposure to helminths and malaria might bias the profile of the antimalarial response toward a T-helper 2 profile or regulatory profile [24]. Another explanation is that individuals susceptible to helminths are more susceptible to malaria. Studies on helminth infections have shown that only a minority of individuals account for the majority of infection burden [47]. This might be due to variation in parasite exposure but could also be due to variation in individual genetic susceptibility [48]. Studies have suggested genetic susceptibility to polyparasitism [49]; hence pregnant women with a genetic susceptibility to hookworm or *M. perstans* infection might be more susceptible to malaria infection. However, adjusting for maternal malaria did not alter the associations between maternal hookworm or *M. perstans* infections and childhood malaria, suggesting that these helminth infections are not simply a marker for genetic susceptibility to malaria.

We used data collected from the EMaBS, a trial that investigated whether anthelminthic treatment during pregnancy could alter the effects of prenatal helminth exposure. Anthelminthic treatment in pregnancy was effective [14], but albendazole and praziquantel had no effect on childhood malaria overall or in subgroup analyses by maternal hookworm and *S. mansoni* infections [35]. This could imply that the association between maternal hookworm and childhood malaria was established early in pregnancy and that single-dose albendazole or praziquantel in the second or third trimester was not sufficient to eliminate or reverse any effect of helminth infection in pregnancy on malaria susceptibility in the offspring. This is particularly likely to be true because this analysis was based on assessment of maternal helminth and malaria status at enrollment to the study, before the trial intervention and intermittent presumptive treatment for malaria were provided to the women (>90% of the mothers received intermittent presumptive treatment for malaria in the second and/or third trimesters, greatly reducing maternal malaria prevalence). It is likely that most of the fetal exposure to maternal malaria occurred before the anthelminthic trial intervention. In contrast, childhood quarterly albendazole was associated with a 15% reduction in the incidence of clinical malaria [35].

Our major limitation was the use of single samples for ascertainment of maternal malaria and helminth infections. Although microscopy is the gold standard in malaria diagnosis, sensitivity is low in pregnancy due to low parasite densities and placental sequestration. In a review [50], the pooled prevalence estimate for peripheral malaria in East and Southern Africa was 32.0% (95% CI 25.9–38.0; n = 11 688), considerably higher than the prevalence we observed. Misclassification of malaria-infected mothers classified as malaria-negative would weaken the strength of observed associations between maternal and childhood malaria. Similarly, for helminths, some mothers may have been misclassified as uninfected. The underestimation of these key exposures may also account for the lack of interaction between maternal malaria and helminths as discussed above. Lastly, we cannot exclude the possibility that confounding by unmeasured covariables could explain some of the observations. Nonetheless, this study had the unique advantage of a prospective birth cohort design, with a large sample size, a long follow-up period, and comprehensive data on potential confounders minimizing residual confounding.

This study provides the first report of an association between helminth infections in pregnancy and malaria in the offspring and suggests that helminth infections in pregnancy may increase the overall burden of childhood malaria in regions of coendemicity. The association between maternal hookworm and childhood malaria was consistent for clinical malaria episodes and asymptomatic parasitemia. However, the mechanism is unclear, and studies are needed to elucidate the significance of maternal helminth infections on fetal and early childhood antimalarial responses. Our findings support the strategy of integrated malaria–helminth control to accelerate the reduction of malaria morbidity and provide pertinent knowledge for the evaluation of malaria vaccine trials because results might be modified by concurrent helminth infections in pregnancy.

## Supplementary Data

Supplementary materials are available at *The Journal of Infectious Diseases* online (http://jid.oxfordjournals.org/). Supplementary materials consist of data provided by the author that are published to benefit the reader. The posted materials are not copyedited. The contents of all supplementary data are the sole responsibility of the authors. Questions or messages regarding errors should be addressed to the author.

Supplementary Data
