# Supplementary material for: Associations Between Maternal Helminth and Malaria Infections in Pregnancy and Clinical Malaria in the Offspring: A Birth Cohort in Entebbe, Uganda
Source: J Infect Dis. 2013 Jul 31;208(12):2007–16. doi: 10.1093/infdis/jit397 (PMC3836463; doi:10.1093/infdis/jit397)
Supplement: Supplementary Data [file supp_208_12_2007__index.html]

Associations Between Maternal Helminth and Malaria Infections in Pregnancy and Clinical Malaria in the Offspring: A Birth Cohort in Entebbe, Uganda — Associations Between Maternal Helminth and Malaria Infections in Pregnancy and Clinical Malaria in the Offspring: A Birth Cohort in Entebbe, Uganda — Supplementary Data 

# Associations Between Maternal Helminth and Malaria Infections in Pregnancy and Clinical Malaria in the Offspring: A Birth Cohort in Entebbe, Uganda

## Supplementary Data

Supplementary Data

**Files in this Data Supplement:**

- Supplementary Data - Docx file
- Supplementary Figure - docx file
