# Supplementary material for: Associations Between Maternal Helminth and Malaria Infections in Pregnancy and Clinical Malaria in the Offspring: A Birth Cohort in Entebbe, Uganda
Source: J Infect Dis. 2013 Jul 31;208(12):2007–16. doi: 10.1093/infdis/jit397 (PMC3836463; doi:10.1093/infdis/jit397)
Supplement: Supplementary Data [file supp_jit397_jit397supp.docx]

The prevalence of infections in pregnant women enrolled in the Entebbe Mother and Baby study.

Hookworm infection was detected in 1,112 of 2,498 women (44.5%), *M. perstans* in 531 of 2,499 (21.2%), *S. mansoni* 458 of 2,498 (18.3%), *Strongyloides* 306 of 2,485 (12.3%), *Trichuris* 226 of 2,498 (9.0%), and Ascaris 58 of 2498 (2.3%). Of the 2,477 tested for all helminth infections, 1,693 (68.3%) had at least one helminth infection (defined as any of the above, Trichostrongylus (26 cases), *Hymenolypsis nana* (4 cases), *Enterobius vermicularis* (1 case) or Loa Loa (1 case)). *Plasmodium falciparum* was detected in 268 of 2,459 women (10.9%) (*P. vivax, ovale* or *malariae* were not detected), HIV in 299 of 2,507 (11.9%) and active syphilis in 18 of 2,507 (1.1%) (110 women had a positive RPR test).
