# Supplementary material for: Associations Between Maternal Helminth and Malaria Infections in Pregnancy and Clinical Malaria in the Offspring: A Birth Cohort in Entebbe, Uganda
Source: J Infect Dis. 2013 Jul 31;208(12):2007–16. doi: 10.1093/infdis/jit397 (PMC3836463; doi:10.1093/infdis/jit397)
Supplement: Supplementary Data [file supp_jit397_jit397supp_fig.docx]

**Figure 1. A flow chart representing the number of mothers enrolled in the EMaBS trial, and the number of children present at follow up.**
